# *TAZ* Is Highly Expressed in Gastric Signet Ring Cell Carcinoma

**DOI:** 10.1155/2014/393064

**Published:** 2014-02-24

**Authors:** Guofeng Yue, Xia Sun, Ana Gimenez-Capitan, Jie Shen, Lixia Yu, Cristina Teixido, Wenxian Guan, Rafael Rosell, Baorui Liu, Jia Wei

**Affiliations:** ^1^The Comprehensive Cancer Centre of Drum Tower Hospital, Medical School of Nanjing University & Clinical Cancer Institute of Nanjing University, 321 Zhongshan Road, Nanjing 210008, China; ^2^Pangaea Biotech, USP Dexeus University Institute, Barcelona, Spain; ^3^Department of General Surgery, Drum Tower Hospital, Medical School of Nanjing University, Nanjing, China

## Abstract

Transcriptional coactivator with PDZ-binding motif (*TAZ*) is known to bind to a variety of transcription factors to control cell differentiation and organ development. We examined *TAZ* protein levels in 146 stage II–IV gastric cancer using immunohistochemistry (IHC), while *TAZ* mRNA was confirmed by quantitative reverse-transcription polymerase chain reaction (QRT-PCR) in 84 samples with enough tissue. *TAZ* protein expression was positive in 113 out of 146 (77.4%) gastric cancer samples. In parallel, *TAZ* mRNA expression was successfully detected in 81 of the 84 (96.4%) samples. Protein levels of *TAZ* were positively correlated with its mRNA levels (*P* = 0.018). High expression of *TAZ* protein was observed with higher percentage in gastric cancer samples with histology of signet ring cell carcinoma (SRCC) than adenocarcinoma (85.7% versus 60.2%, *P* = 0.001). Similarly, *TAZ* mRNA level was higher in SRCC than in adenocarcinoma (*P* = 0.003). When correlated with survival, the median overall survival (OS) is 14 months (95% CI: 12.2–15.8 months) in all patients. There was no significant association between survival and other clinical characteristics or *TAZ* expression levels. Our results show that *TAZ* is highly expressed in SRCC. *TAZ* might be considered as a target for the treatment of gastric SRCC in future.

## 1. Introduction

Gastric cancer is the fourth most common cancer with the leading causes of cancer death in East Asian countries and some western countries [1, 2]. Signet ring cell carcinoma (SRCC) is characterized by cells with abundant mucin in the cytoplasm and nuclei located at the cell periphery. This type of carcinoma appears to be relatively frequent in women and young patients [3, 4]. It has long been thought to have a worse prognosis than other forms of gastric cancer. Recently, studies have begun to question this idea. Several studies find that the survival of patients with early SRCC was not significantly different from other types of gastric carcinoma [5]. This was because SRCC of the stomach is less likely to lymph node metastasis and it had a higher proportion in the early stage of gastric carcinoma than other carcinomas [6, 7]. The genetic background of SRCC has rarely been investigated, and the molecular basis of their growth, differentiation, and metastasis still remains unclear. Therefore, studies of the molecular profile of gastric SRCC and identification of new molecular markers are both relevant to improve the diagnosis and the prognosis of the tumor.

Transcriptional coactivator with PDZ-binding motif (*TAZ*), also called WW-domain containing transcription regulator 1 (*WWTR1*), has been defined for its role in the nucleus [8–10]. It functions directly as a transcriptional regulator by interacting with several nuclear factors and plays a central role in the Hippo pathway, which regulates the size and shape of organ development [8–12]. *TAZ *was described as controlling gene important for muscle differentiation, lung and respiratory epithelia differentiation, cardiac and limb development, adipogenesis and osteogenesis, and tumorigenesis. Most human tissues, except thymus and peripheral blood leucocytes, express *TAZ* mRNA, with the highest levels in kidney, heart, placenta, and lung [8–12]. *TAZ* has been identified as an oncogene and has an important role in tumorigenicity of many cancers, such as non-small cell lung cancer [13, 14], papillary thyroid carcinoma [15], and colon cancer [16]. They found that *TAZ* gene expression signature was over-represented in poorly differentiated tumors compared with well-differentiated low-grade tumors. Importantly, *TAZ* confers cancer stem cell-related traits in breast cancer cells [17–19], further highlighting its importance in tumor initiation and progression. According to present studies, *TAZ* is significantly associated with poor survival of cancer, so *TAZ *may be a novel prognostic indicator for cancer progression. But so far, no report has been published concerning the relationships between *TAZ* expression and clinicopathological features and prognosis of gastric cancer patients. Therefore, the objectives of this study were to evaluate the relationships between *TAZ* expression and the clinicopathological parameters of gastric cancer and to evaluate its potential role as a prognostic biomarker and an anticancer target.

## 2. Materials and Methods

146 gastric samples were collected from patients of the Comprehensive Cancer Center, Drum Tower Hospital Affiliated to Medical School of Nanjing University, from November 2007 to August 2011. All samples have been pathologically proven to be cancer. *TAZ* protein levels were examined by IHC in 146 samples. Meanwhile, *TAZ* mRNA levels were confirmed by quantitative reverse-transcription polymerase chain reaction (QRT-PCR) in 84 samples with enough tissue. This project has been approved by Institutional Review Board of Drum Tower Hospital.

### 2.1. Immunohistochemical Staining for* TAZ*

After dewaxing in xylene and rehydrating stepwise in ethanol, sections were subjected to heat-induced antigen retrieval. The endogenous peroxidase activity was inactivated in a solution containing 3% hydrogen peroxide (H_2_O_2_) in methanol. In the negative control, the primary antibody was omitted. Skeletal muscle was used as positive control. Pretreated sections were incubated with rabbit polyclonal *TAZ* antibody (T3467, 1 : 50, Epitomics) at 4°C overnight, followed by secondary antibody. Immunohistochemical staining was evaluated independently by two pathologists without knowledge of patient characteristics, and discrepancy was resolved by consensus review. Tissue was scored (H score) based on the total percentage of positive cells ((≤5%) = 0, (6%~25%) = 1, (26%~50%) = 2, (51%~75%) = 3, and (>75%) = 4) and the intensity of the staining (0, 1, 2, or 3), where *H* is the percentage of positive score multiply intensity score. The sample was considered negative if *H* = 0 and positive if *H* was more than 0. Positive samples were also categorized as weak (1+) if *H* = 1 to 4, middle (2+) if *H* = 5 to 8, and strong (3+) if *H* was more than 8 [20]. A minimum of 100 cells were evaluated in calculating the *H* score. Patients with negative or weak staining were considered as lower group, while patients with middle and strong staining were considered as higher group.

### 2.2. Quantitative Reverse-Transcription Polymerase Chain Reaction (QRT-PCR) Assessment of *TAZ* Expression

Three 5 *μ*m sections were prepared from FFPE tumor blocks that contained at least 80% tumor cells. After hematoxylin-eosin staining, RNA was isolated in accordance with a proprietary procedure as we published before [21]. Briefly, paraffin was removed by xylene, and macrodissected tissues were lysed in a proteinase K-containing buffer at 60°C for 16 h. RNA was purified by phenol and chloroform extractions followed by precipitation with isopropanol in the presence of sodium acetate at −20°C. The RNA pellet was washed in 70% ethanol and resuspended in RNase-free water followed by DNase. M-MLV Reverse Transcriptase Kit (Ambion, Carlsbad, CA) was used to generate cDNA for quantitative reverse-transcription polymerase chain reaction (QRT-PCR) to detect the expression of *β*-actin (used as endogenous control) and *TAZ*. Commercial human total RNA was used for each RT reaction as calibrator. Template cDNA was amplified with specific primers and probes (Table 1) for *β*-actin and *TAZ* using TaqMan Universal Master Mix (Applied Biosystems, Foster City, CA). The QRT-PCR was performed to quantify gene expression using ABI Prism 7900HT Sequence Detection System (Applied Biosystems, Foster City, CA). The PCR conditions were 50°C for 2 min and 95°C for 15 min, followed by 40 cycles at 95°C for 15 sec and 60°C for 1 min. Relative gene expression quantifications were calculated according to the comparative Ct method [21] and analyzed with the Applied Biosystems analysis software. *TAZ* mRNA levels were further divided into three groups according to tercile levels.

### 2.3. Statistical Analysis

Correlations between *TAZ* protein expression and clinicopathological parameters were analyzed by *χ* ^2^ test. Correlations between TAZ protein expression and mRNA were also analyzed by *χ* ^2^ test. The Mann-Whitney *U* test and the Kruskal-Wallis test were used to test the associations between *TAZ* mRNA levels and clinical characteristics. Survival curves were assessed by the Kaplan-Meier method. Two-sided *P* < 0.05 was considered statistically significant. All analyses were performed with the SPSS 17.0 software package (SPSS Inc., Chicago, USA).

## 3. Results

A total of 111 males and 35 females were included with ages ranging from 24 to 92 years (median, 61 years). Eighty-three patients (56.8%) with the histology of adenocarcinoma and 63 patients (43.2%) were confirmed as signet ring cell carcinoma. There were 6 patients (4.1%) with stage II (4 stage IIA and 2 stage IIB), 136 patients (93.2%) with stage III (22 stage IIIA, 36 stage IIIB, and 80 stage IIIC), and 4 patients (2.7%) with stage IV disease. 40 patients received 5-FU and/or oxaliplatin-based chemotherapy. The median follow-up time was 14.3 months (95% CI = 2.53 to 27.5 months).

### 3.1. Relationship between *TAZ* Protein Expression and mRNA Expression

**Figure 1:**
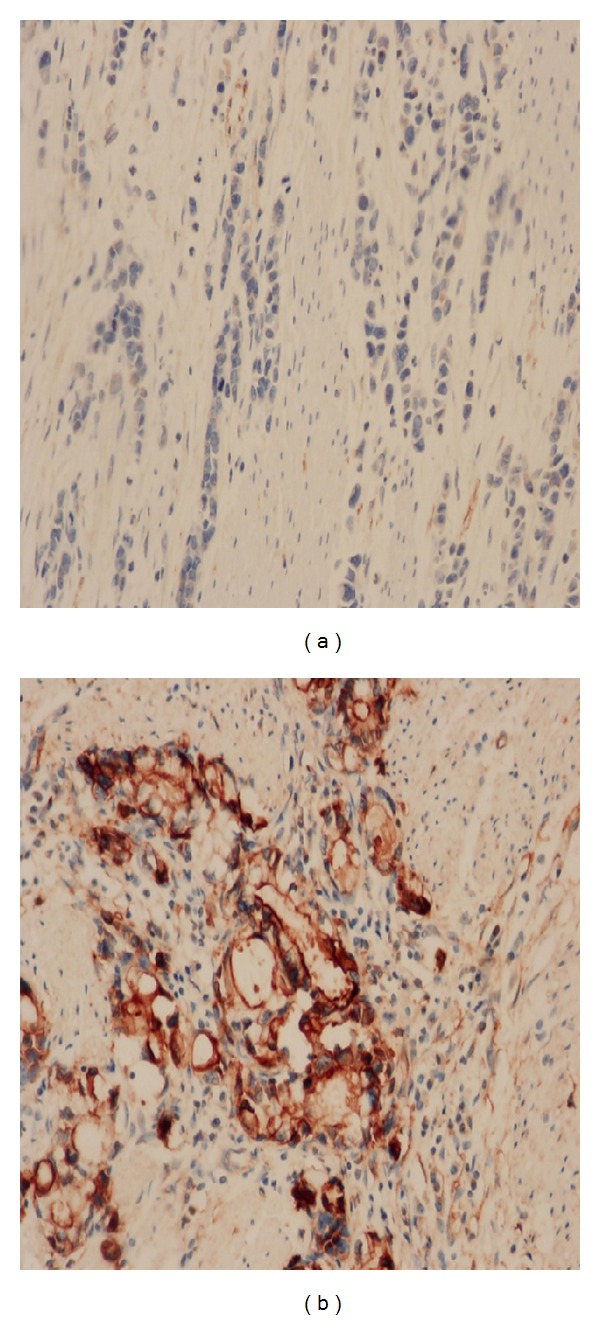
TAZ protein expression in gastric cancer. (a) Negative case at high magnification (×400). (b) Positive case (*H* score = 12) with signet ring cell phenotype at high magnification (×400).

*TAZ* protein expressions were positive in 113 of 146 (77.4%) samples. *TAZ* had nuclear and cytoplasmic expression (Figure 1). In parallel, 84 samples had enough tissue to detect *TAZ* mRNA. *TAZ* mRNA expression was found in 81 of the 84 (96.4%) samples. In* TAZ *mRNA low expression group, 44.8% of patients had low level of *TAZ* protein. Protein levels of *TAZ* were correlated with its mRNA levels (*P* = 0.018). There were 88.5% of patients with high *TAZ* protein levels in *TAZ* mRNA high group, 76.9% of patients with high *TAZ* protein levels in mRNA intermediate group, and 55.2% of patients with high *TAZ* protein levels in mRNA low group (Table 2).

### 3.2. Relationship between* TAZ* Protein Expression and Clinicopathological Characteristics

*TAZ* protein levels were higher in SRCC than in adenocarcinoma (*P* = 0.001) and higher in Grade 3 cancer than in Grade 2 cancer (*P* = 0.004). However, there was no difference between *TAZ* protein levels and age (*P* = 0.294), gender (*P* = 0.376), tumor site (*P* = 0.159), lymph node metastasis (*P* = 0.232), or stage (*P* = 0.785) (Table 3).

### 3.3. Relationship between mRNA Expression and Clinicopathological Characteristics

*TAZ* mRNA level in signet ring cell carcinoma was higher than adenocarcinoma (median levels: 4.64 versus 2.02, *P* = 0.003). However there was no difference between *TAZ* mRNA levels and patients' age (*P* = 0.374), gender (*P* = 0.696), tumor site (*P* = 0.069), lymph node metastasis (*P* = 0.899), p-TNM stage (*P* = 0.492), or histological grade (*P* = 0.375) (Table 3).

### 3.4. Survival for Gastric Cancer Patients According to *TAZ* Protein and mRNA Levels

The median overall survival (OS) is 14 months (95% CI = 12.2 to 15.8 months) in all patients. The median OS is longer in younger patients (16.9 months, 95% CI = 11.9–22.6 months) than in elder patients (12.4 months, 95% CI = 9.3–14.9). Patients with stage II had a longer OS (23.25 months, 95% CI = 10.4–36.1 months) than stage III (14 months, 95% CI = 12.4–15.7 months) and stage IV (5.1 months, 95% CI = 4.6-5.6 months). There was no significant association between OS and gender (*P* = 0.652), tumor site (*P* = 0.312), differentiation (*P* = 0.477), lymph node metastasis (*P* = 0.294), *TAZ* protein levels (*P* = 0.481), or *TAZ* mRNA levels (*P* = 0.132) (Table 4).

## 4. Discussion

The Hippo pathway plays an important role in cell proliferation, organ size control, and cancer development and progression. *TAZ* is a transcriptional coactivator that is inhibited by Hippo pathway [22, 23]. Aberrant inactivation of the Hippo pathway and/or overexpression of *TAZ* results in transcriptional activation of their downstream targets. *TAZ* overexpression induces cell proliferation and epithelial-mesenchymal transition (EMT) and inhibits apoptosis and contact inhibition [24, 25]. EMT is a process in which cells lose epithelial-like characteristics, such as cell-cell adhesion and polarity, and acquire mesenchymal properties that include increased motility. Most carcinomas exhibit a partial EMT, which is thought to promote the formation of cell populations that are enriched in cancer stem cells (CSCs). Cordenonsi et al. [19] found that *TAZ* was required to sustain self-renewal of breast CSCs and to induce their tumorigenic potential. And most interestingly, *TAZ* was overrepresented in poorly differentiated breast tumors compared with well-differentiated ones. *TAZ* protein levels increase during EMT and that this is required for mammosphere formation, which is also promoted by EMT. Bhat et al. [26] found that *TAZ* expression was lower in proneural glioblastomas (GBMs) and lower grade gliomas compared with GBMs that had a mesenchymal phenotype. *TAZ* expression in GBMs is positively correlated with the expression for mesenchymal genes and is also predictive of poor overall survival. Moreover, *TAZ* is significantly associated with poor survival of colon cancer patients in two independent colon cancer datasets, comprising 522 patients [16]. In present study, we successfully detected and compared *TAZ* protein and mRNA expressions in gastric tumor tissues (Table 2) and correlated *TAZ* levels with clinicopathological parameters and survival (Table 3).

We also found that *TAZ* was higher expressed in SRCC than adenocarcinoma in either protein or mRNA levels (Table 3). SRCC has long been thought to have a worse prognosis than other forms of gastric cancer. Recently, SRCC has been known to have different biologic characteristics between early stage and advanced stage gastric cancer. In early gastric cancer, SRCC has been reported to have better prognosis than others because of less lymph node metastasis and a more grossly depressed type, which is helpful for diagnosis. However, in advanced gastric cancer, SRCC has been characterized to be a more grossly infiltrative type, although the reason is still unclear. Few molecular markers had been proven to have relationship with SRCC, such as the M2 isoform of pyruvate kinase (*PKM2*), bone morphogenetic proteins (*BMP-7*), and transcriptional factor forkhead box P3 (*FoxP3*). *PKM2* was identified as a driver of aerobic glycolysis and has been shown to be the isoform preferentially overexpressed in tumor cells. Well and moderately differentiated adenocarcinoma showed significantly higher expression of *PKM2* than SRCC. *PKM2* protein expression was found to negatively correlate with survival in SRCC patients [27]. *BMP-7* is signaling molecule belonging to the transforming growth factor (TGF) superfamily. Recent studies demonstrated that *BMP-7* expression is found in various human cancers and regulates cell differentiation, proliferation, migration, invasion, and apoptosis [28]. *BMP-7* expression was significantly higher in the differentiated histology group than in the undifferentiated group. And the *BMP-7 *positive group had significantly poorer survival than the *BMP-7* negative group in the undifferentiated group. The key role of *FoxP3* is induction of immunesuppressive function to maintain self-tolerance. It is widely accepted that *FoxP3* is expressed not only in mice and humans but also in tumor cells such as melanoma stomach and might have relationship with immunosuppressive effect. Yoshii, et al [29] demonstrated that *FoxP3* was expressed in SRCC. *FoxP3* would allow them to escape from immune surveillance, thereby resulting in cancer progression such as lymph node metastasis. But the molecular pathogenesis of SRCC remains largely unknown. In present study, we find that *TAZ* expression was higher in SRCC than in adenocarcinoma for the first time. We hypothesize that *TAZ* might participate in tumorigenesis and development of signet ring cells. However, future studies were more needed. Our results show the way for future studies aiming to reveal additional insights into the molecular mechanisms of signet ring cell. Since *TAZ* was reported to bind to a variety of transcription factors to control cell differentiation and organ development, such as *p73* (p53 family member), *Runx2* (runt family member 2), *PPAR* *γ* (peroxisome prolif-erator-activated receptor *γ*), *TTF-1* (thyroid transcription factor-1), *Pax3* (paired box 3), *Tbx5* (T-box 5), *Smad2/3/4* (SMAD family member 2/3/4), and *TEAD* [8–10]. In present study, the *TAZ* protein is mainly accumulated in the nucleus with a less cytoplasmic presence. *TAZ* might be considered as a novel target for the treatment of gastric cancer, especially in SRCC. However, in the present study, the sample size is rather limited and the distribution between different stages is also scattered, which might be the reason that we did not find any correlations between *TAZ* and stage or prognosis. Further studies with larger number of patients were warranted to valid utility of *TAZ *in gastric cancer patients.

## Figures and Tables

**Table 1:** Primers and probes of *TAZ * and *β*-actin.

Primer	*TAZ *	*β*-actin
Forward primer	5′ CCAGTGCCTCAGAGGTCCA 3′	5′ TGAGCGCGGCTACAGCTT 3′
Reverse primer	5′ ATCTGCTGCTGGTGTTGGTG 3′	5′ TCCTTAATGTCACGCACGATTT 3′
Probe	**6FAM** 5′ CCAAATCTCGTGATGAAT 3′** MGB**	**6FAM** 5′ ACCACCACGGCCGAGCGG 3′** TAMRA**

**Table 2:** *TAZ* protein levels and mRNA levels.

*TAZ* mRNA	Protein low expression (IHC: 0-1+)	Protein high expression (IHC: 2+-3+)	*P*
Low expression	13 (44.8%)	16 (55.2%)	0.018
Intermediate expression	6 (23.1%)	20 (76.9%)	
High expression	3 (11.5%)	23 (88.5%)	

**Table 3:** The relationship between *TAZ* and clinicopathological characteristics.

Characteristics	*TAZ *protein levels		*P*	*TAZ* mRNA levels	*P*
	low IHC (0~1+)	High IHC (2+~3+)			
Age			0.294		0.374
<60	15 (24.2%)	47 (75.8%)		4.45 ± 4.84	
≥60	27 (32.1%)	57 (67.9%)		3.92 ± 4.93	
Sex			0.376		0.696
Female	8 (22.9%)	27 (77.1%)		4.71 ± 6.44	
Male	34 (30.6%)	77 (69.4%)		3.99 ± 4.32	
Histology			0.001		0.003
Adenocarcinoma	33 (39.8%)	50 (60.2%)		3.15 ± 3.25	
SRCC	9 (14.3%)	54 (85.7%)		6.71 ± 7.02	
Tumor site			0.159		0.069
Distal stomach	13 (26.5%)	36 (73.5%)		5.43 ± 6.02	
Proximal stomach	17 (41.5%)	24 (58.5%)		2.2 ± 2.31	
Whole stomach	11 (22.9%)	37 (77.1%)		4.37 ± 4.82	
Unknown	1 (12.5%)	7 (87.5%)		2.94 ± 1.51	
Lymph node			0.232		0.899
N0-1	8 (40%)	12 (60%)		4.8 ± 6.99	
N2-3	34 (27%)	92 (73%)		4.04 ± 4.45	
Stage			0.785		0.492
II	1 (16.7%)	5 (83.3%)		3.60 ± 2.30	
III	40 (29.4%)	96 (70.6%)		4.24 ± 5.05	
IV	1 (25%)	3 (75%)		2.80 ± 2.59	
Histological grade			0.004		0.375
G2	16 (48.5%)	17 (51.5%)		4.05 ± 5.86	
G3	26 (23%)	87 (77%)		4.19 ± 4.54	

**Table 4:** The median overall survival for patients according to *TAZ* levels.

Characteristics	Number of patients	Median overall survival (months) (95% CI)	*P*
Age			0.029
<60	61 (41.8%)	16.9 (11.9–22.6)	
≥60	85 (58.2%)	12.4 (9.3–14.9)	
Sex			
Female	35 (24.0%)	14.8 (8.3–21.4)	
Male	111 (76.0%)	14.0 (12.6–15.5)	
Tumor site			0.312
Distal stomach	49 (33.6%)	14.1 (10.4–17.9)	
Proximal stomach	40 (27.3%)	13.7 (12.8–14.6)	
Whole stomach	49 (33.6%)	14.1 (9.8–18.3)	
Unknown	8 (5.5%)	6.7 (4.8–8.5)	
Lymph node			0.294
N0~1	20 (13.7%)	18.1 (8.7–27.5)	
N2~3	126 (86.3%)	14.0 (12.2–15.8)	
Stage			0.029
II	6 (4.1%)	23.25 (10.4–36.1)	
III	136 (93.2%)	14.0 (12.4–15.7)	
IV	4 (2.7%)	5.1 (4.6–5.6)	
Histological grade			0.477
G2	33 (22.6%)	12.9 (10.1–15.7)	
G3	113 (77.4%)	14.2 (11.9–16.5)	
*TAZ* protein expression			0.481
Low expression	42 (28.8%)	13.7 (10.9–16.5)	
High expression	104 (71.2%)	14.1 (11.4–16.7)	
*TAZ* mRNA expression			0.132
Low expression	29 (35.8%)	8.1 (5.2–10.9)	
Intermediate expression	26 (32.1%)	14.0 (12.6–15.5)	
High expression	26 (32.1%)	9.6 (6.5–12.6)	
